# A novel variant of fructose‐1,6‐bisphosphatase gene identified in an adult with newly diagnosed hepatitis C

**DOI:** 10.1002/jmd2.12256

**Published:** 2022-02-17

**Authors:** Helena Fawdry, Rebecca Gorrigan, Radha Ramachandran, William M. Drake

**Affiliations:** ^1^ St Bartholomew's Hospital London UK; ^2^ Royal London Hospital London UK; ^3^ Guy's and St Thomas' London UK

**Keywords:** FBP1 gene, fructose‐1,6‐bisphosphatase deficiency, gluconeogenesis, hepatitis C, hypoglycaemia, inborn errors of fructose metabolism

## Abstract

Hepatic fructose‐1,6‐bisphosphatase (FBPase) deficiency commonly presents with acute crises during infancy when glycogen stores are depleted. In these patients, dependence on glycogenolysis means that the duration of normoglycaemia is related to liver glycogen stores. Clinical hallmarks of FBPase deficiency include hypoglycaemia and lactic acidosis with or without ketosis. Patients commonly present with hyperventilation, vomiting, tachycardia, reduced consciousness and glucagon‐resistant hypoglycaemia. Between crises, patients are usually well with normal growth and development; however significant ingestion of fructose, sucrose or glycerol during acute crises may be fatal, hence the importance of a prompt diagnosis. We present the case of a 30‐year‐old male who presented to our tertiary centre acutely unwell, shortly following a diagnosis of hepatitis C, which we speculate may have precipitated this severe presentation. He had similar, milder episodes throughout childhood. Furthermore, a pathological homozygous sequence variant in fructose‐1,6‐bisphosphatase (*FBP1*) gene, previously unreported, was identified. Diagnosis in adulthood is underreported in the literature, however, represents an important, albeit rare, cause of hypoglycaemia and lactic acidosis.

**SYNOPSIS**

*FBP1* deficiency is an important, albeit rare, cause of hypoglycaemia and lactic acidosis in adults; we speculate that this severe presentation was precipitated by newly diagnosed hepatitis C.

## 1 INTRODUCTION

Deficiency of hepatic fructose‐1,6‐bisphosphatase (FBPase), a rate‐limiting gluconeogenic enzyme, is a rare autosomal recessive disorder that commonly presents with acute crises in neonates and infants.^1^, ^2^, ^3^ FBPase is encoded by the *FBP1* gene, which consists of seven coding exons and is located at chromosome 9q22.3.^4^ During the initial period of fasting, normoglycaemia is maintained by hepatic glycogenolysis, thus the period for which an individual may safely fast is proportional to the quantity of available hepatic glycogen.^1^ Following prolonged fasting, there is an increased reliance on gluconeogenesis from substrates such as lactate, pyruvate, alanine, and glycerol, with the rate‐limiting conversion of fructose 1,6‐bisphosphate into fructose 6‐phosphate catalysed by FBPase.^1^, ^5^ Consequently, affected patients commonly present with hypoglycaemia when glycogen stores are limited or exhausted, such as when fasting or during catabolism associated with inter‐current illnesses, particularly during the neonatal period.^5^

Acute crises are characterised by hyperventilation, vomiting, episodic irritability, tachycardia, somnolence and hypotonia, and may be fatal, especially in neonates and infants.^2^, ^6^ Biochemical hallmarks include glucagon‐resistant hypoglycaemia, elevated serum lactate with associated acidosis, increased lactate/pyruvate ratio and pseudohypertriglyceridaemia (secondary to elevated glycerol levels).^5^ Serum ketone levels, including 3‐hydroxybutyrate and acetoacetate, may either be elevated or normal.^5^, ^6^ Between crises, patients are usually well with normal growth and development; generally, frequency of attacks decreases with age due to increasing tolerance to catabolic stressors.^1^, ^2^, ^5^

Here, we report a case of an acute presentation associated with newly diagnosed hepatitis C, in a patient with suspected FBPase deficiency. Furthermore, a novel sequence variant, previously undescribed in the literature, was identified in this patient.

## 2 CASE REPORT

A 30‐year‐old male born to non‐consanguineous parents presented to our emergency unit with symptomatic hypoglycaemia (point‐of‐care venous glucose 1.8 mmol/L, laboratory value 2.0 mmol/L). He reported 2 days of mild, intermittent abdominal pain and frequent food craving, not responsive to fruit, simple carbohydrates, or dairy products. Despite oral intake, he developed dysarthria, hyperhidrosis, somnolence, and asthenia, with subsequent onset of severe epigastric pain and vomiting immediately prior to presentation. He denied any blurred vision, dizziness, or seizures. There were no infective or episodic symptoms of note.

He had been recently diagnosed with hepatitis C, discovered incidentally during investigations for exertional chest pain with associated lactataemia. He denied recent alcohol intake although reported significant stress related to this diagnosis. His medication history was significant only for branded multivitamins (ReVision^©^ and Centrum^©^). He denied any use of prescription, recreational or over‐the‐counter medications, including psychotropic or anxiolytic medications.

Throughout childhood, he had multiple hospital admissions due to protracted vomiting during intercurrent illness, with associated Mallory‐Weiss tears. On one such occasion, he recalled being hypoglycaemic and requiring intravenous glucose, and on another required a blood transfusion. These episodes persisted into adulthood, and he had identified a maximum fasting period of 15 h, with symptoms readily responsive to sugary foods. Unfortunately, no unifying diagnosis had been made, and these episodes were instead managed symptomatically. His family history was significant for Type 2 diabetes mellitus and hypothyroidism. Furthermore, he reported that his paternal aunt and father had similar, albeit less severe, symptoms that had not been investigated and were self‐managed.

On examination, he was pale, waxy, tachycardic and hypertensive. There was no evidence of any organomegaly, oral mucosal hyperpigmentation, café au lait spots, or peripheral stigmata of chronic liver disease. He was of normal height and neurocognitive development.

Emergency room investigations showed a severe lactic acidosis (lactate 18 mmol/L, pH 6.9) and marked leucocytosis (neutrophils 17 × 10^9^/L [RR: 2–7 × 10^9^/L], monocytes 1.2 × 10^9^/L [RR: 0.2–0.8 × 10^9^/L]) with a normal C‐reactive protein (4 mg/L [RR: <5 mg/L]). He was hyperkalaemic (6.5 mmoL/L [RR 3.9–5.3 mmol/L]), without concurrent acute kidney injury (serum creatinine 96 μmol/L [RR: 45–84 μmol/L]). Liver function testing demonstrated an acutely elevated ALT (163 unit/L [RR: 0–41unit/L]), with normal bilirubin and synthetic function. Of note, recent transient elastography (FibroScan^©^) performed during hepatitis workup showed only mild fibrosis (5.5 kPa). No infective focus was found on bacteriology specimens (blood, urine) or imaging (CT abdomen and pelvis) and toxicology screen was negative. Due to persistent acidosis and hyperkalaemia despite intravenous crystalloid and dextrose therapy, he required haemofiltration, following which he remained euglycaemic without intravenous dextrose.

He was referred to our unit and underwent a supervised fast, developing symptomatic hypoglycaemia after 18 h. His serum glucose was 1.1 mmol/L with low insulin (<1 mU/L) and C‐peptide (50 pmol/L). He had significant ketosis (serum beta‐hydroxybutyrate 2.36 mmol/L), as well as elevated free fatty acids (3.16 mmol/L), urate (645 μmoL/L) and lactate (10.4 mmoL/L). His basal pituitary profile was normal.

A clinical diagnosis of fructose‐1,6‐bisphosphatase deficiency was made and he was referred for genetic analysis and expert inherited metabolic disorders (IMD) advice. The Illumina HiSeq platform was used to sequence coding regions and splicing sites of 30 glycogen storage disorders/gluconeogenesis/glycogen synthesis genes using TruSight One Panel target enrichment system (Illumina). A homozygous previously unreported variant of unknown significance affecting a highly conserved nucleotide in the splice donor region of intron 1 of the *FBP1* gene was identified. In silico analysis predicted this to affect splicing, and clinical and biochemical findings were consistent with pathogenicity. Unfortunately, it was not possible to arrange genotyping of this individual's family members due to geographical constraints. Fructose‐1,6‐phosphatase activity in white cells was subsequently also found to be very low (7 nmol/h/mg ptn, [RR 101–463 nmol/h/mg ptn]), thus confirming pathogenicity of the novel mutation. He was initiated on appropriate management, centred on avoidance of fasting, regular meals which include slowly absorbed carbohydrates, and uncooked cornstarch (UCCS) before bed as cover for overnight fast. Unfortunately, he was unable to tolerate the UCCS due to gastrointestinal discomfort. Therefore, he was encouraged to take a late‐night snack high in low glycaemic index (GI) carbohydrates and given an oral emergency regimen (ER) of dissolvable 25% glucose polymer (50 g/sachet to be dissolved in 200 mL of water) sachets to be used when unable to eat and drink normally. He was advised to have a low threshold for presenting to the emergency department for intravenous glucose if unable to tolerate, or not improving with, the oral emergency regimen.

He completed a 12 week course of hepatitis eradication therapy consisting of elbasvir with grazoprevir (Zepatier^©^). He achieved sustained virological response and had a normal liver ultrasound at 12‐month follow‐up. Following treatment completion, he experienced one further hypoglycaemic episode precipitated by a stressful personal event, which was easily terminated with one sachet of oral ER. He once again denied concurrent use of psychotropic or recreational drugs at this time. It was not possible to deduce whether fasting tolerance had increased, due to a change in the pattern of dietary intake following patient education.

## 3 DISCUSSION

Despite being an important part of the differential diagnosis for an adult presenting with lactic acidosis and hypoglycaemia, cases of FBPase deficiency diagnosed in adulthood are poorly described in the literature, with only 4 case reports identified during literature search of all English‐language papers.^7^, ^8^, ^9^, ^10^ Nearly half of all affected patients present in the first 4 days of life with an acute crisis, secondary to deficient glycogen stores.^2^, ^11^ Many of those patients diagnosed in adulthood present multiple times, including during childhood, before a unifying diagnosis is made.^9^, ^10^ Whilst the preservation of glycolysis contributes to reduced clinical severity, undiagnosed *FBP1* deficiency is potentially fatal, particular if fructose, sucrose or glycerol containing intravenous solutions are used to manage acute crises or ensuing complications such as cerebral oedema.^6^, ^12^ Whilst not common first‐line agents for hypoglycaemia, a timely and accurate diagnosis remains essential to reduce associated morbidity and mortality.^13^ If the patient survives these acute episodes, development generally progresses normally, with an increased tolerance to fasting with aging, thought to be secondary to increasing hepatic glycogen content and decreased intercurrent infections.^14^ Of note, however, despite optimal glycaemic control, one patient diagnosed in adulthood still developed early onset cognitive impairment and sensorineural deafness.^9^

All case reports identified in the literature pertain to non‐European patients, relevant due to differences in disease‐causing genetic variants of *FBP1* gene between geographical regions.^13^, ^15^ Over 100 pathogenic variants have been reported to date, of which around one‐quarter are frameshift variants (*n* = 25) and around one‐third are missense variants (*n* = 35).^16^ As described above, analysis revealed a pathogenic homozygous mutation in the splice donor region of intron 1 of the *FBP1* gene, *c.170+4A>G,p.(?)*, a novel variant previously undescribed in the literature^17^, ^18^ (see Table 1).^13^, ^15^ Three other pathogenic splice region variants of *FBP1* gene have been recorded, affecting introns 4, 5 and 7.^16^, ^19^, ^20^, ^21^

**TABLE 1:** Summary table detailing clinical timeline of key events and interventions

Age	Presentation	Interventions
6 months–15 years	Severe vomiting resulting in Mallory‐Weiss tears during intercurrent illnesses At least once, found to be profoundly hypoglycaemic	IV glucose Total parental nutrition (TPN) Blood transfusion
6 months–30 years	Neuroglycopenia associated with poor oral intake	Easily terminated with teaspoon of sugar
30 years	Severe neuroglycopenia following recent incidental diagnosis of Hepatitis C Ketotic lactic acidosis Hyperkalaemia	Standard hyperkalaemia management (insulin‐dextrose, calcium chloride 10%) 30 mL sodium bicarbonate 8.4% Haemofiltration
>30 years	Mild glucopenia	Easily terminated with oral ER Hepatitis C eradication therapy with elbasvir with grazoprevir

We speculate that this acute, severe presentation was precipitated by recently diagnosed hepatitis C. FBPase catalyses the hydrolysis of fructose 1,6‐bisphosphate to fructose 6‐phosphate, a common reaction to both the Calvin cycle and gluconeogenesis.^22^ Whilst phosphorylation of fructose 6‐phosphate in glycolysis utilises ATP, there is no reciprocal production during gluconeogenesis.^22^ Therefore, under conditions of low cellular ATP concentrations, FBPase is relatively inactive, thus avoiding a ‘futile cycle’ in which ATP is consumed with no metabolic gain.^22^ As a result of active consumption of ATP to facilitate viral RNA replication, cells infected with hepatitis C virus (HCV) display a significantly reduced concentration of ATP in in vitro studies.^23^

Furthermore, maintenance of normoglycaemia during fasting in individuals with FBPase deficiency is closely related to liver glycogen availability.^1^ HCV‐mediated upregulation of DPP‐IV expression resulted in a significant reduction in serum GLP‐1 in one single‐centre case‐control study, relevant due to GLP‐1's role in stimulating glycogen formation.^24^, ^25^ Additionally, HCV can inactivate *Akt*, an important protein kinase involved in glycogen synthase activation, another integral glycogenesis enzyme.^24^ On the contrary, however, mice studies failed to show a difference in hepatic glycogen stores between HCV‐affected and control specimens, thus further research is required to confirm or refute our hypothesis.^26^

Guidelines published by British Inherited Metabolic Disease Group in 2008 suggest the avoidance of fructose and sucrose during acute illness.^27^ There is one reported case of an 8‐year‐old in whom a metabolic decompensation was precipitated following a fructose‐based cough syrup.^28^ However, other studies report patients tolerate fructose up to 2 g/kg in divided doses throughout the day and hence the need for restriction of fructose and sucrose in diet, particularly in older children and adults when well, is less clear.^5^ Our patient was advised to adopt an unrestricted balanced diet which includes fruits when well, and fructose and sucrose avoidance when unwell. Approximately one‐third of patients in a consensus report were managed with UCCS to extend overnight fasting.^29^ Of these patients, two‐thirds (*n* = 23) reported a fasting tolerance of greater than 10 h when well.^29^ In this case report, at 12‐month follow up, the patient was unfortunately unable to tolerate UCCS and instead adopted a carbohydrate‐rich late‐night snack, with no further early morning hypoglycaemic episodes reported.

Despite the novelty of our case in European literature, and thus its benefit in advancing the evidence base, the authors recognise several important limitations. First, given that this is a single case report, it is not possible to draw any causal relationship between newly diagnosed hepatitis C and acute crisis. Furthermore, the avoidance of fasting as an important means to prevent hypoglycaemic episodes limits conclusions regarding the impact of hepatitis C eradication therapy on prolonging fasting interval.

## 4 CONCLUSION

FBPase deficiency, a key gluconeogenesis enzyme, is a rare autosomal recessive disorder that commonly presents acutely in early childhood when glycogen stores are limited or exhausted. The relationship between hepatic impairment, subsequent impact on glycogen stores, and precipitation of inborn errors of metabolism represents an important area of future study.

Despite being underreported in the literature, up to 40% of patients under the care of Adult IMD services are diagnosed in adulthood.^30^ Given the potential for this diagnosis to be missed in earlier life, this therefore represents an important differential in patients presenting with hypoglycaemia and lactic acidosis.

## CONFLICT OF INTEREST

The authors declare no potential conflict of interests.

## AUTHOR CONTRIBUTIONS

All authors had direct involvement in the patient's clinical care, at resident or attending level, and thus acquisition of data. All authors were involved in the planning of the paper. Helena Fawdry wrote the first draft of the manuscript and all other authors provided extensive input into further drafts and all approved the final manuscript.

## PATIENT CONSENT

Informed consent was obtained from this patient to be included in the case report.

## ETHICS STATEMENT

No experimentation or research was involved that required informed consent and no patient identifying data are included.

## Data Availability

My manuscript has no associated data.
